# NOL11, Implicated in the Pathogenesis of North American Indian Childhood Cirrhosis, Is Required for Pre-rRNA Transcription and Processing

**DOI:** 10.1371/journal.pgen.1002892

**Published:** 2012-08-16

**Authors:** Emily F. Freed, José-Luis Prieto, Kathleen L. McCann, Brian McStay, Susan J. Baserga

**Affiliations:** 1 Department of Genetics, Yale University School of Medicine, New Haven, Connecticut, United States of America; 2 Centre for Chromosome Biology, School of Life Sciences, National University of Ireland Galway, Galway, Ireland; 3 Department of Molecular Biophysics and Biochemistry, Yale University School of Medicine, New Haven, Connecticut, United States of America; 4 Department of Therapeutic Radiology, Yale University School of Medicine, New Haven, Connecticut, United States of America; University of Louisville, United States of America

## Abstract

The fundamental process of ribosome biogenesis requires hundreds of factors and takes place in the nucleolus. This process has been most thoroughly characterized in baker's yeast and is generally well conserved from yeast to humans. However, some of the required proteins in yeast are not found in humans, raising the possibility that they have been replaced by functional analogs. Our objective was to identify non-conserved interaction partners for the human ribosome biogenesis factor, hUTP4/Cirhin, since the R565W mutation in the C-terminus of hUTP4/Cirhin was reported to cause North American Indian childhood cirrhosis (NAIC). By screening a yeast two-hybrid cDNA library derived from human liver, and through affinity purification followed by mass spectrometry, we identified an uncharacterized nucleolar protein, NOL11, as an interaction partner for hUTP4/Cirhin. Bioinformatic analysis revealed that NOL11 is conserved throughout metazoans and their immediate ancestors but is not found in any other phylogenetic groups. Co-immunoprecipitation experiments show that NOL11 is a component of the human ribosomal small subunit (SSU) processome. siRNA knockdown of NOL11 revealed that it is involved in the cleavage steps required to generate the mature 18S rRNA and is required for optimal rDNA transcription. Furthermore, abnormal nucleolar morphology results from the absence of NOL11. Finally, yeast two-hybrid analysis shows that NOL11 interacts with the C-terminus of hUTP4/Cirhin and that the R565W mutation partially disrupts this interaction. We have therefore identified NOL11 as a novel protein required for the early stages of ribosome biogenesis in humans. Our results further implicate a role for NOL11 in the pathogenesis of NAIC.

## Author Summary

Ribosomes are the cellular factories that produce proteins. Making a ribosome is a complex and energy intensive process that requires hundreds of different factors. Ribosome biogenesis is an essential process, and therefore mutations that partially disrupt this process lead to disease. One such disease is North American Indian childhood cirrhosis (NAIC), which is caused by a mutation in a ribosome biogenesis protein called hUTP4/Cirhin. We looked for proteins that interact with hUTP4/Cirhin, since we hypothesized that disruption of this interaction could play a role in the development of NAIC. We identified a novel protein called NOL11, which is only found in animals and not in any other organisms. We showed that NOL11 is required for ribosome biogenesis and acts at one of the earliest steps in this process. We then showed that NOL11 interacts with the region of hUTP4/Cirhin that contains the NAIC mutation and that the NAIC mutation interferes with the interaction between hUTP4/Cirhin and NOL11. Further study of the interaction between hUTP4/Cirhin and NOL11 will give insight into the development of NAIC, as well as elucidate some of the differences in ribosome biogenesis between animals and other organisms.

## Introduction

Ribosome biogenesis is one of the most fundamental of cellular processes. It is so important for cell growth that in a HeLa cell, 7500 ribosomal subunits are made every minute [1] and in eukaryotes, 60% of total cellular transcription is devoted to ribosome biogenesis [2]. Ribosome biogenesis occurs in the nucleolus and begins, in human cells, when RNA polymerase I (Pol I) transcribes the pre-ribosomal RNA (pre-rRNA) as a 47S polycistronic precursor. The pre-rRNA then undergoes multiple cleavage and chemical modification events before giving rise to the mature 18S, 5.8S, and 28S rRNAs [reviewed in 3]. The cleavages that free the mature 18S small ribosomal subunit rRNA are mediated by a large ribonucleoprotein particle called the small subunit (SSU) processome, which contains over 70 proteins and the U3 small nucleolar RNA (snoRNA) [4], [5]. The SSU processome assembles cotranscriptionally on the pre-rRNA [6].

The majority of the hundreds of nucleolar proteins involved in ribosome biogenesis were first identified in yeast [reviewed in 3], [7] and were subsequently found in the nucleoli of human cells (http://www.lamondlab.com/NOPdb3.0) [8]. While most of these proteins and their functions are conserved to humans [5], [9], it is becoming clear that important differences exist in how ribosome biogenesis is regulated between yeast and humans. In particular, database searches using HomoloGene (http://www.ncbi.nlm.nih.gov/homologene) and BKL PROTEOME (http://www.biobase-international.com) reveal that approximately 10% of ribosome biogenesis factors, about half of them essential, are not conserved between these two organisms, raising the possibility that they are replaced by functional analogs.

Bioinformatic and biochemical analyses have shown that proteins in the SSU processome exist as subcomplexes prior to assembly into the SSU processome [10]–[12]. One of these subcomplexes, the t-UTP/UTPA subcomplex, is required for both optimal pre-rRNA transcription and processing in yeast [13] and in human cells [14]. In yeast, multiple biochemical methods were used to arrive at a consensus of the seven members of the t-Utp/UtpA subcomplex: Utp4, Utp5, Utp8, Utp9, Utp10, Utp15, and Utp17 [10], [11], [13]. Of the t-Utp subcomplex members, only five are conserved to humans: hUTP4/Cirhin, hUTP5, hUTP10, hUTP15, and hUTP17 [14]. Two additional human SSU processome proteins, not in the t-UTP/UTPA subcomplex, 1A6/DRIM (ortholog of yUtp20) [15] and hALP (ortholog of yKre33) [16], have recently been shown to be required for optimal pre-rRNA transcription in human cells, in contrast to yeast where they are only required for pre-rRNA processing.

What does it mean to be a t-UTP? In both yeast [13] and in human cells [14], the t-UTPs are required for the SSU processome-mediated cleavages that liberate the mature 18S rRNA from the polycistronic pre-rRNA precursor, as determined by Northern blot analysis. They also immunoprecipitate rDNA chromatin (ChIP), and are required for optimal transcription of the rDNA, as demonstrated by transcription run-on analysis. In yeast, the t-Utp subcomplex has additionally been shown to assemble in the absence of either rDNA transcription or of the U3 snoRNA. Taken together, these results suggest that the t-UTPs form a subcomplex in the absence of active ribosome biogenesis, are members of the SSU processome, and also have the additional function of regulating rDNA transcription. Interestingly, while yUtp4 is required for both optimal pre-rRNA transcription and processing in yeast [13], [17], hUTP4/Cirhin is not required for optimal pre-rRNA transcription in human cells but is required for pre-rRNA processing [14]. It is unclear why this difference exists but it may be related to the lack of complete conservation of all proteins in this subcomplex from yeast to humans.

Several years ago, a missense mutation (R565W) in a t-UTP, hUTP4/Cirhin, was reported to cause North American Indian childhood cirrhosis (NAIC) [18]. NAIC is a familial cholestasis that begins with transient neonatal jaundice and then progresses to biliary cirrhosis. Liver transplantation is the only effective therapy [19]. Although hUTP4/Cirhin is known to be required for ribosome biogenesis, little is known about the molecular mechanism(s) that leads to this disease.

We have previously shown using yeast as a model system that loss of interaction between yUtp4 and another t-Utp/UtpA subcomplex member, yUtp8, disrupts ribosome biogenesis and leads to cell death [17]. yUtp8 is one of the rare SSU processome proteins that is not conserved from yeast to humans, leading to the hypothesis that there may be a functional analog that replaces it. Furthermore, disruption of the interaction between hUTP4/Cirhin and this as yet unidentified protein may play a role in the etiology of NAIC.

We therefore set out to identify interaction partners for hUTP4/Cirhin using yeast two-hybrid (Y2H) methodology. We identified a previously uncharacterized, metazoan-specific protein, nucleolar protein 11 (NOL11) [20]. In this study, we show that NOL11 is a member of the human t-UTP subcomplex of the SSU processome and, as such, is required for both optimal pre-rRNA transcription and processing. It is additionally required for proper nucleolar morphology. Finally, we show that NOL11 interacts with the region of hUTP4/Cirhin that contains the NAIC mutation, and that the NAIC mutation reduces NOL11 interaction in the context of a C-terminal hUTP4/Cirhin fragment. This provides support for a defective hUTP4/Cirhin-NOL11 interaction underlying the pathogenesis of NAIC.

## Results

### hUTP4/Cirhin interacts with NOL11

**Figure 1:**
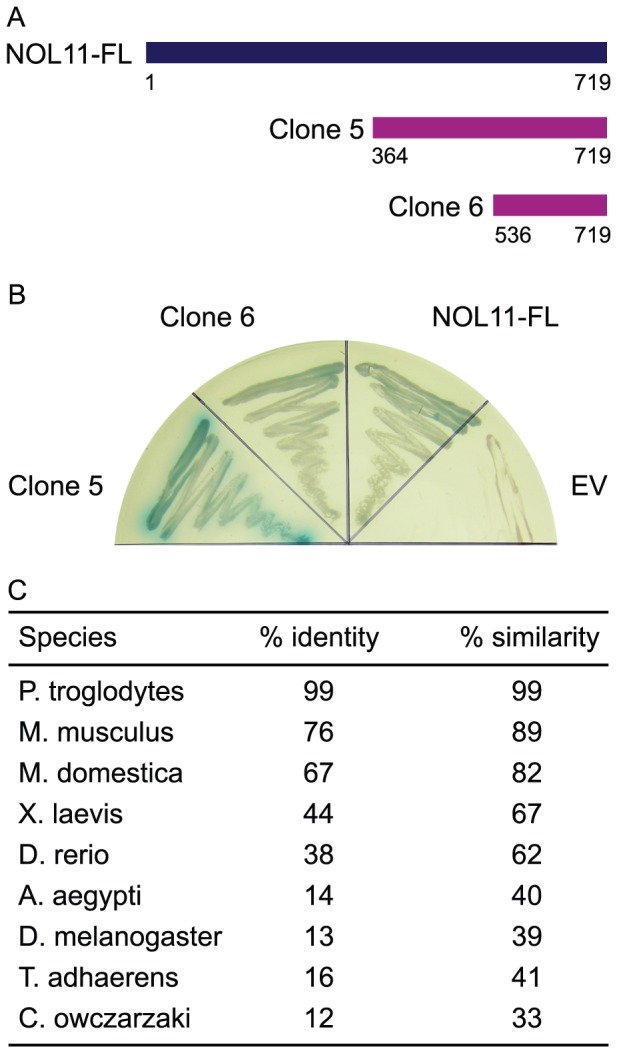
hUTP4/Cirhin interacts with NOL11. (A) hUTP4/Cirhin was screened against a human liver cDNA library using the yeast two-hybrid methodology. Two positive clones were recovered (clones 5 and 6), both of which were C-terminal fragments of nucleolar protein 11 (NOL11). (B) Directed Y2H analysis confirms that hUTP4/Cirhin interacts with the two positive clones as well as with full-length NOL11 (NOL11-FL). EV is the empty Y2H prey vector. Growth of a blue yeast colony indicates interaction. (C) NOL11 is conserved throughout metazoans. BLAST searches using human NOL11 (NP_056277.2) as a query were used to identify NOL11 orthologs in other species. BioEdit [57] was used to calculate percent identity and similarity.

To discover interaction partners for hUTP4/Cirhin, two approaches were taken. First, a yeast two-hybrid (Y2H) cDNA library derived from human liver was screened using hUTP4/Cirhin as a bait. Two clones were recovered, both of which encoded C-terminal fragments of nucleolar protein 11 (NOL11; NP_056277.2; Figure 1A and 1B). Clone 5 contained NOL11 amino acid residues 364–719 and clone 6 contained amino acid residues 536–719 (full-length NOL11 is 719 amino acids long). Directed Y2H analysis using the recovered clones and full-length NOL11 confirmed that NOL11 indeed interacts with hUTP4/Cirhin. NOL11 was first identified in a proteomic analysis of the human nucleolus in HeLa cells [20] and was subsequently also identified in proteomic analyses of the human T-cell nucleolus [21] and in WI-83 cell and NB4 cell nucleoli [8]. The Conserved Domain Database predicts that NOL11 contains the nucleolar specific domain, NUC205 [22], but has no known structural or functional domains [23]. Although NOL11 has been localized to the nucleolus in previous studies, its function has not yet been elucidated. NOL11 contains no sequence similarity to Utp8 and therefore could not have been discovered in BLAST searches using Utp8 as a query.

In order to determine how well NOL11 is conserved to other species, the human NOL11 protein sequence was used as a query for a BLAST search (http://blast.ncbi.nlm.nih.gov/Blast.cgi). Figure 1C shows that NOL11 is highly conserved in mammals and is also present in lower animals, although NOL11 orthologs could not be identified outside of the Metazoan kingdom. A putative NOL11 homolog was also identified in *Capsaspora owczarzaki*, a single-celled eukaryote that is a candidate for being the evolutionary link between single-celled animals and metazoans [24], [25]. Taken together, these results suggest that NOL11 is likely functionally important in metazoans.

To find interaction partners that may have been missed in the Y2H screen, hUTP4/Cirhin was Strep- and HA-tagged in HEK-293 cells. Proteins associated with hUTP4/Cirhin were affinity purified and identified by mass spectrometry (Table 1). Only proteins that were identified in both biological duplicates and had at least five unique peptides identified between these duplicates were considered to be associated with hUTP4/Cirhin. Importantly, mass spectrometry revealed NOL11 as a protein associated with hUTP4/Cirhin, giving independent confirmation of the Y2H analysis results. Furthermore, four out of five of the previously known t-UTP subcomplex members were identified as being in the same complex as NOL11: hUTP4/Cirhin, hUTP5, hUTP15, and hUTP17. A full list of identified proteins is available in Table S1.

**Table 1:** Proteins that co-purify with hUTP4/Cirhin identified by mass spectrometry.

Gene name	Alias	Swissprot accession	Unique peptides in experiment 1	Unique peptides in experiment 2
hUTP4/Cirhin+	CIRH1A	Q969X6	44	40
hUTP5	WDR43	Q15061	13	11
NOL11		Q9H8H0	8	4
hUTP15		Q8TED0	7	3
ACACA*		Q13085	6	8
hUTP17	WDR75	Q8IWA0	3	4

Only proteins that were identified in both biological duplicates and had at least five unique peptides identified between duplicates are included. The full list can be found in Table S1.

+ Bait protein used for affinity purification.

* Proteins that are repeatedly isolated with other bait proteins.

### NOL11 is a t-UTP

**Figure 2:**
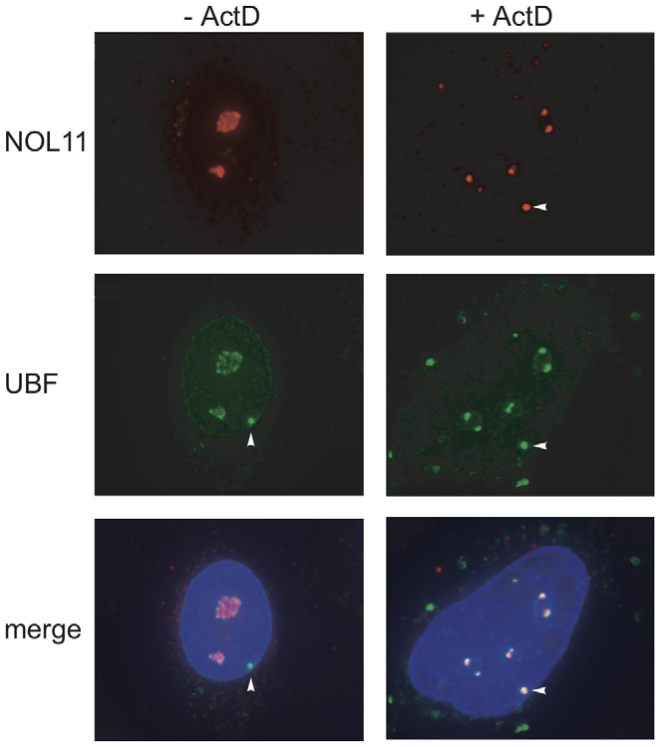
NOL11 localization is dependent on RNA polymerase I transcription. NOL11 was detected in HT1080-3D1 cells using an anti-NOL11 antibody. An anti-UBF antibody was used to stain nucleoli and pseudo-NORs. Co-localization of NOL11 and UBF is shown in the merged image. DAPI was used to stain chromatin. Cells were either not treated (−ActD) or treated (+ActD) with 0.1 µg/ml ActD for 1 h before fixation. Blue = DAPI, Red = NOL11, Green = UBF. Pseudo-NORs are indicated with arrowheads.

Given that NOL11 interacts with hUTP4/Cirhin and associates with the known human t-UTPs, it is likely that NOL11 functions in ribosome biogenesis. All of the known t-UTPs are nucleolar and also localize to a punctate structure called a pseudo-nucleolar organizing region (pseudo-NOR) [26]. Pseudo-NORs were created by introducing *Xenopus* Enhancer (*XEn*) elements into human cells, which serves to sequester the Pol I transcription machinery and Pol I-associated proteins [27]. Although NOL11 was originally identified in proteomic analyses of human nucleoli [8], [20], [21], its subcellular localization has not been studied directly. Therefore, we determined the localization pattern of NOL11 in the pseudo-NOR-containing cell line, HT1080-3D1. Endogenous NOL11 was detected in HT1080-3D1 cells by immunofluorescence microscopy using an anti-NOL11 antibody. An antibody against Upstream Binding Factor (UBF), a known nucleolar protein, was used to co-stain nucleoli. Co-staining with the two antibodies showed a high degree of overlap between NOL11 and UBF, confirming that NOL11 localizes to the nucleolus (Figure 2). The subcellular localization of NOL11 was also determined after treatment with Actinomycin D (ActD), a known inhibitor of Pol I transcription. In the absence of transcription, NOL11 localized to nucleolar caps and became enriched in pseudo-NORs (Figure 2). Localization to nucleolar caps after ActD treatment has been observed for many other components of the SSU processome and Pol I subunits [28], [29].

**Figure 3:**
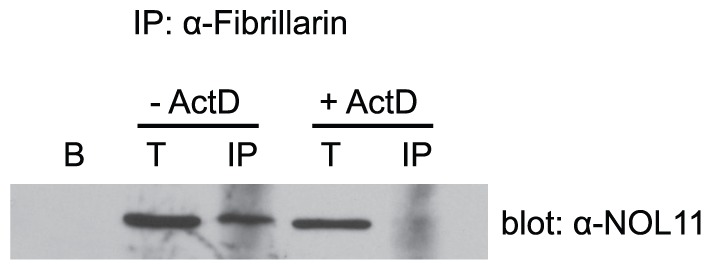
RNA polymerase I transcription is required for NOL11 association with the SSU processome. HeLa cells were lysed and the lysate was clarified by centrifugation. The known SSU processome protein, fibrillarin, was immunoprecipitated using an anti-fibrillarin antibody (72B9) conjugated to beads. Total (5%) and immunoprecipitated proteins were separated by SDS-PAGE and transferred to a PVDF membrane. Association of NOL11 with the SSU processome was assayed by Western blotting for NOL11 with an anti-NOL11 antibody. Cells were either not treated (−ActD) or treated (+ActD) with 0.1 µg/ml ActD for 1 h before harvesting. B = beads alone, T = total protein, IP = immunoprecipitation.

We hypothesized that NOL11 is a component of the SSU processome based on its nucleolar localization and interaction with hUTP4/Cirhin. Co-immunoprecipitation (co-IP) using an antibody directed against fibrillarin (mouse monoclonal 72B9) [30], an SSU processome protein that is part of the U3 snoRNP subcomplex, revealed that NOL11 associates with fibrillarin (Figure 3). This suggests that NOL11 is incorporated into the SSU processome. Furthermore, this association is dependent on Pol I transcription, as NOL11 no longer immunoprecipitates fibrillarin after treatment with ActD (Figure 3).

**Figure 4:**
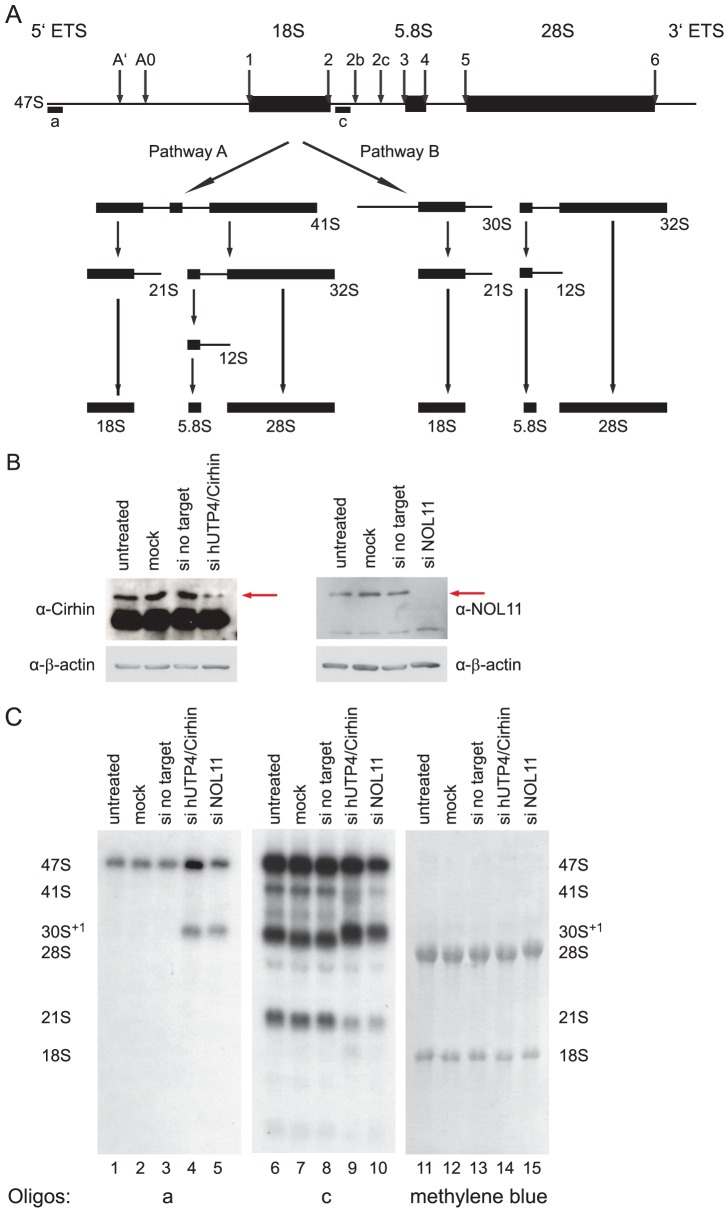
NOL11 is required for pre-rRNA processing in HeLa cells. (A) Diagram of pre-rRNA processing in human cells. Cleavage sites are indicated with labeled arrows. The positions of oligonucleotide probes used for Northern blotting are indicated with lettered lines. (B) hUTP4/Cirhin and NOL11 were successfully knocked down by siRNAs. After siRNA treatment, total protein was extracted from HeLa cells, separated by SDS-PAGE, and transferred to a PVDF membrane. Western blotting was used to check for the presence of hUTP4/Cirhin (left) or NOL11 (right). (C) Northern blot analysis of pre-rRNA processing. After siRNA treatment, total RNA was extracted from HeLa cells, separated on an agarose/formaldehyde gel, and transferred to a Hybond XL nylon membrane. RNA was detected with the indicated oligonucleotide probe or with methylene blue.

Given that NOL11 is a component of the SSU processome, it is likely that it functions in pre-rRNA processing. Furthermore, since it co-purifies with the human t-UTP subcomplex, it may also be required for optimal pre-rRNA transcription. To determine at which stage(s) NOL11 is involved in ribosome biogenesis, siRNAs were used to knock down NOL11 in HeLa cells. hUTP4/Cirhin, which has previously been shown to be required for pre-rRNA processing in HeLa cells [14], was also knocked down by siRNA for comparison. Knockdown efficiency was determined by Western blot with anti-NOL11 or anti-hUTP4/Cirhin antibodies. NOL11 was depleted to non-detectable levels after 4 days and hUTP4/Cirhin expression was reduced after 2 days (Figure 4B). Total RNA was extracted from cells before and after siRNA treatment and pre-rRNA processing was assayed by Northern blot using the indicated oligonucleotide probes (Figure 4A and 4C). In agreement with previous studies [14], knockdown of hUTP4/Cirhin led to an accumulation of the 30S^+1^ pre-rRNA (Figure 4C, compare lanes 1–3 to lane 4) indicating a failure of SSU processome mediated cleavages at sites A', A0, 1, and 2b. The same accumulation of the 30S^+1^ pre-rRNA was also observed in NOL11 knockdown cells (Figure 4C, compare lanes 1–3 to lanes 4 & 5). The lack of processing can also be seen by the shift in mobility of the 30S^+1^ species in hUTP4/Cirhin and NOL11 knockdown cells (Figure 4C, compare lanes 6–8 to lanes 9 & 10). In both hUTP4/Cirhin and NOL11 knockdown cells, the increase in the 30S^+1^ pre-rRNA was accompanied by a concomitant decrease in the 21S pre-rRNA (Figure 4C, compare lanes 6–8 to lanes 9 & 10). These results show that NOL11 is required for pre-rRNA processing at sites A', A0, 1, and 2b.

Unlike hUTP4/Cirhin, when NOL11 is knocked down, a general reduction in all pre-rRNA processing intermediates was observed (Figure 4C, compare lanes 6–9 to lane 10). Consistent with this, quantitation of the intensity of the 47S pre-rRNA band in the blot probed with oligonucleotide c showed an approximately 30% decrease in 47S pre-rRNA levels in NOL11 siRNA-transfected versus mock-transfected cells. A reduction in all pre-rRNAs was previously observed upon depletion of any of the t-Utps in *Saccharomyces cerevisiae* [13] and, by analogy, suggests that NOL11 may be required for optimal transcription of the rDNA in human cells.

**Figure 5:**
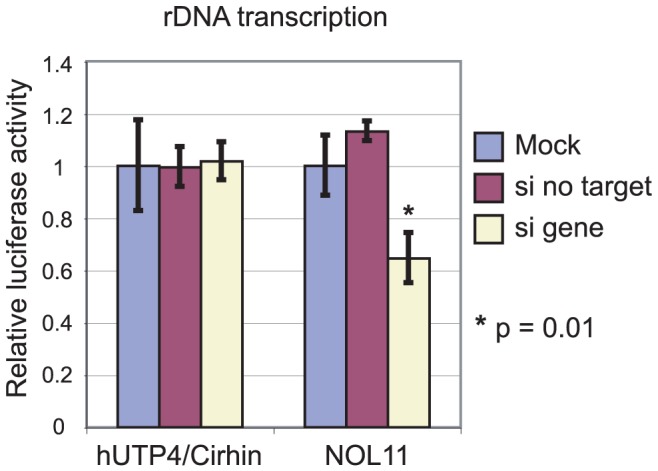
NOL11 is required for optimal rDNA transcription. Cells were transfected with the indicated siRNA and a plasmid containing firefly luciferase under the control of the rDNA promoter (pHrD-IRES-Luc). Transfection efficiency was normalized by co-transfection with a plasmid containing *Renilla* luciferase. Luciferase levels were measured using a 20/20n luminometer (Turner BioSystems) and the Dual Luciferase Assay System (Promega) 24 h after transfection. Each experiment was performed in triplicate. Average relative luciferase activity is shown with the standard deviation indicated by the error bars.

To test this hypothesis, a reporter gene was used to assay Pol I transcription. The pHrD-IRES-Luc plasmid, which contains luciferase under the control of the human rDNA promoter [31], has previously been successful for detecting changes in rDNA transcription [16], [32], [33]. Either hUTP4/Cirhin or NOL11 was knocked down in HeLa cells and Pol I transcription was assayed by transfection with pHrD-IRES-Luc. A second plasmid containing *Renilla* luciferase under the control of an RNA polymerase II promoter [34] was co-transfected to control for transfection efficiency. Luciferase activity was measured 24 h post-transfection. In agreement with previous results [14], knockdown of hUTP4/Cirhin did not have an effect on rDNA transcription (Figure 5). In contrast, when NOL11 was knocked down, a 40–50% decrease in rDNA transcription was observed (Figure 5), indicating that NOL11 is required both for optimal rDNA transcription and pre-rRNA processing, as are other members of the t-UTP subcomplex [13], [14].

### Loss of NOL11 causes abnormal nucleolar morphology

**Figure 6:**
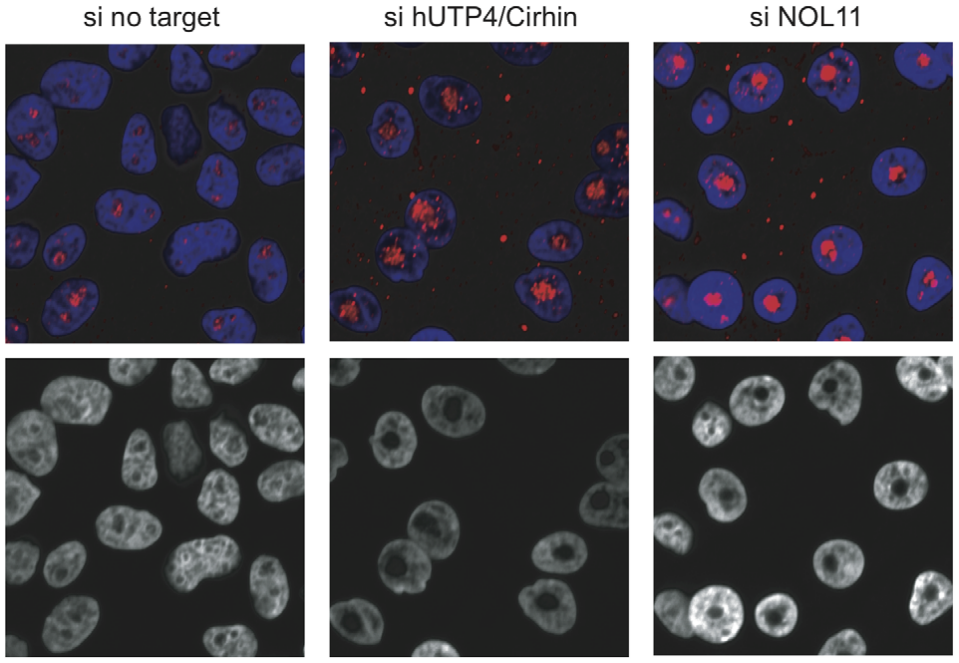
Loss of NOL11 causes abnormal nucleolar morphology. The indicated siRNAs were transfected into MCF-10A cells and nucleolar morphology was determined 72 h later by staining nucleoli with an anti-fibrillarin antibody (72B9; top panels). Hoechst was used to stain chromatin. Images from the blue channel only are shown in the bottom panels. Blue = Hoechst, Red = fibrillarin.

The nucleolus assembles around rDNA that is being actively transcribed and, furthermore, nucleolar structure is affected by changes in ribosome biogenesis [35], [ and reviewed in 36]. Similarly, ActD treatment of cells leads to nucleolar segregation/disintegration [28], [37]. Therefore, we evaluated if NOL11 plays a role in determining nucleolar morphology by asking whether there were changes after siRNA knockdown of NOL11 or hUTP4/Cirhin in MCF-10A human mammary epithelial cells. Nucleolar morphology was probed 72 h after siRNA transfection by staining cells with an anti-fibrillarin antibody. While cells treated with a non-targeting control siRNA contained 1–4 nucleoli per cell, cells that were treated with siRNAs against hUTP4/Cirhin or NOL11 contained only one, very large nucleolus (Figure 6). These larger nucleoli may be the result of chromosome coalescing [38]. The change in nucleolar size and shape after knockdown of NOL11 further supports a role for NOL11 in ribosome biogenesis.

### Interaction between hUTP4/Cirhin and NOL11 requires sequences that contain the NAIC mutation

**Figure 7:**
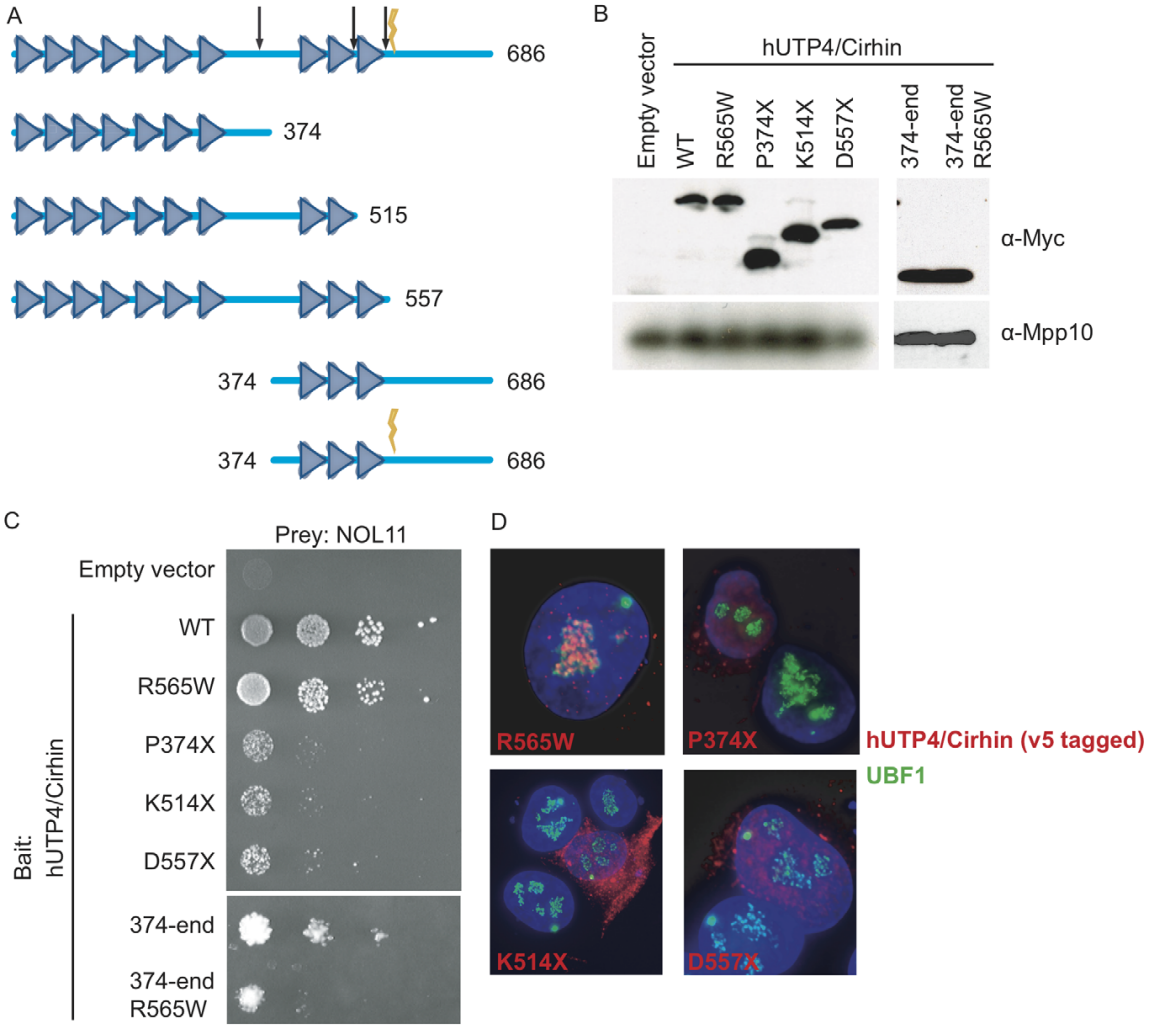
The NAIC mutation disrupts the interaction between NOL11 and a C-terminal fragment of hUTP4/Cirhin. (A) Diagram of the motifs in hUTP4/Cirhin predicted by the SMART database [61] and the hUTP4/Cirhin mutants used in this study. Triangles represent WD40 repeats. The lightning bolt indicates the NAIC mutation (R565W) and the arrows show the locations of truncations at P374, K514, and D557. (B) Western blot showing expression of MYC-tagged hUTP4/Cirhin wild type (WT) and mutants (R565W, P374X, K514X, D557X, 374-end, 374-end R565W) from the Y2H bait vector. An anti-Mpp10 antibody was used to assess gel loading. (C) Y2H analysis between NOL11 and hUTP4/Cirhin mutants shows that NOL11 interacts with the C-terminus of hUTP4/Cirhin but not with the C-terminus of hUTP4/Cirhin containing the NAIC mutation. 10-fold serial dilutions were spotted onto medium lacking leucine, tryptophan, and histidine; growth on this medium indicates an interaction. (D) Immuno-fluorescence microscopy showing the subcellular localization of hUTP4/Cirhin mutants. V5-tagged hUTP4/Cirhin mutants were transfected into HT1080-3D1 cells and detected with an anti-V5 antibody. An anti-UBF antibody was used to co-stain nucleoli. DAPI was used to stain chromatin. Blue = DAPI, Red = hUTP4/Cirhin, Green = UBF.

NOL11 is a previously uncharacterized protein that interacts with hUTP4/Cirhin and is required for pre-rRNA processing and transcription. We therefore hypothesized that loss, or partial loss, of interaction between hUTP4/Cirhin and NOL11 could disrupt ribosome biogenesis and lead to the pathophysiology of NAIC. To test this, the NAIC mutation (R565W), several C-terminal truncations (P374X, K514X, and D557X), an N-terminal deletion (374-end), and an N-terminal deletion containing the NAIC mutation (374-end R565W) were introduced into hUTP4/Cirhin (Figure 7A). Each construct was cloned into a Y2H bait vector (MYC-pGBK) and expression was confirmed by Western blot with an anti-MYC antibody (9E10 ascites; Figure 7B) [39].

Y2H analysis showed that hUTP4/Cirhin containing the NAIC mutation was still able to interact with NOL11 (Figure 7C). In contrast, when hUTP4/Cirhin was truncated by 162 amino acids or more (D557X, K514X and P374X mutants), yeast did not grow as well on selective media, indicating a partial loss of interaction with NOL11 (Figure 7C). Y2H analysis between NOL11 and an N-terminal deletion of hUTP4/Cirhin (374-end mutant) confirmed that NOL11 does indeed interact with the C-terminus of hUTP4/Cirhin. This region contains the sequences of hUTP4/Cirhin that are mutated in NAIC. To determine if the NAIC mutation would have a deleterious effect in the context of a shorter hUTP4/Cirhin construct, we introduced the NAIC mutation into the 374-end mutant (374-end R565W). The results show that yeast expressing both hUTP4/Cirhin 374-end R565W and NOL11 do not grow as well on selective media as yeast expressing hUTP4/Cirhin 374-end without the mutation and NOL11. This indicates that the NAIC mutation interferes with the interaction between hUTP4/Cirhin and NOL11 in the context of the C-terminus of hUTP4/Cirhin. Interestingly, the interaction between NOL11 and hUTP4/Cirhin may be required for proper subcellular localization of hUTP4/Cirhin, since hUTP4/Cirhin mutants that do not interact with NOL11 also fail to localize to the nucleolus (Figure 7D).

## Discussion

In this study, we have characterized NOL11 as a novel, metazoan-specific protein involved in ribosome biogenesis. NOL11 is localized to the nucleolus, the site of ribosome biogenesis, and also associates with proteins in the SSU processome, the large ribonucleoprotein particle that is required for maturation of the small ribosomal subunit 18S rRNA. Furthermore, when NOL11 is knocked down in human cell lines, aberrant pre-rRNA processing, a reduction in rDNA transcription, and abnormal nucleolar morphology are observed. These findings all support a role for NOL11 in ribosome biogenesis.

We have established that NOL11 is involved in ribosome biogenesis, but is it a t-UTP? Affinity purification and mass spectrometry of hUTP4/Cirhin-associated proteins place NOL11 in a complex that contains four out of the five known human t-UTPs, strongly suggesting that NOL11 is indeed a t-UTP. Additionally, after ActD treatment, NOL11 is sequestered with the Pol I transcription machinery in pseudo-NORs. Interestingly, NOL11 is not found in pseudo-NORs during active rDNA transcription, which suggests that in the absence of rDNA transcription NOL11 is in excess and therefore is able to interact with pseudo-NORs. It could also suggest that NOL11 is only involved in active transcription, since pseudo-NORs are transcriptionally silent, even in the absence of ActD [27]. Not only does NOL11 associate with the Pol I transcription machinery and the other t-UTPs, but it co-immunoprecipitates fibrillarin, demonstrating that NOL11 is able to associate with the SSU processome. As in yeast [13], this association with the SSU processome only occurs in the presence of rDNA transcription. siRNA knockdown followed by Northern blot analysis confirms that NOL11 is required for SSU processome mediated cleavages of the pre-rRNA. siRNA knockdown of NOL11 also results in a 40–50% reduction in rDNA transcription, showing that NOL11 has dual roles in both optimal pre-rRNA processing and transcription, which is expected for a t-UTP.

Since NOL11 is a t-UTP that interacts with hUTP4/Cirhin, it is a likely candidate for being the functional analog of Utp8. NOL11 and Utp8 are similarly sized proteins with 719 and 713 amino acid residues, respectively. However, neither NOL11 nor Utp8 have any known domains, precluding a comparison of function by bioinformatic analysis. We have shown that NOL11 interacts with the C-terminus of hUTP4/Cirhin, similar to the interaction between Utp8 and Utp4 in yeast, supporting the conclusion that NOL11 and Utp8 are, in fact, functional analogs. If NOL11 and Utp8 truly are functional analogs, we would expect that they might share other conserved protein-protein interactions [40], [41]. In yeast, Utp8 also interacts with Utp9, which is, strikingly, the other member of the yeast t-Utp subcomplex that is not present in humans. Therefore, Utp9 is likely also replaced by a functional analog in human cells. In yeast, Utp9 interacts with Utp8 and Utp15 by Y2H [17]; consequently using either hUTP15 or NOL11 as a bait for a Y2H library screen or for affinity purification would likely identify the functional analog of Utp9, if it exists.

A growing number of proteins that are required for optimal transcription of the rDNA in humans are being identified. However, the mechanism by which these proteins regulate Pol I transcription has not been completely elucidated. In yeast, the t-Utps associate with short, stable transcripts comprising the first 125–138 nt of the 35S pre-rRNA [Gallagher and Baserga, unpublished data]. Furthermore, t-Utp binding to the pre-rRNA is likely one of the earliest steps of ribosome biogenesis, since the t-Utp subcomplex is needed for the rest of the SSU processome to assemble [11]. Association with both the rDNA chromatin and the 5′ end of the pre-rRNA physically places the t-Utps in a position where they can easily be involved in both pre-rRNA transcription and processing.

An alternate role for the t-Utp subcomplex has been suggested in yeast. Wery et al. propose that the t-Utp subcomplex is required for stability of the pre-rRNA rather than for transcription [42]. In direct opposition to the results presented in Gallagher et al. [13], when Wery et al. deplete a t-Utp subcomplex member and then perform transcription run-on (TRO) analysis, they do not observe a decrease in rDNA transcription. It is unclear why these two sets of results are in conflict with each other. However, the TRO results presented in Gallagher et al. are further supported by the observation that fewer nascent pre-rRNA transcripts are present in EM visualizations of chromatin spreads after depletion of a t-Utp in yeast. Furthermore, TRO in human cell lines also confirms a role for the t-UTP subcomplex in regulating rDNA transcription [14]. More recently, the pHrD-IRES-Luc reporter has been used in human cell lines to demonstrate that a subset of SSU processome proteins is required for optimal rDNA transcription [this study], [ 15,16]. Therefore, it seems likely that the proteins themselves, as well as their function in regulating rDNA transcription, are largely conserved from yeast to humans.

We identified NOL11 through its interaction with hUTP4/Cirhin, the protein that is mutated in North American Indian childhood cirrhosis (NAIC). Several previous high throughput Y2H analyses, including one liver-specific analysis, either did not include hUTP4/Cirhin as a bait or failed to identify proteins that interact with hUTP4/Cirhin [43]–[45]. However, one other interaction partner for hUTP4/Cirhin has been reported. Yu et al. [46] screened a human liver cDNA library using hUTP4/Cirhin as a bait and recovered Cirip as an interacting protein. Cirip is a nuclear protein that stimulates transcription of the HIV-1 LTR enhancer element. The lack of nucleolar localization of Cirip suggests that the interaction between Cirip and hUTP4/Cirhin is unlikely to play a role in ribosome biogenesis.

In another previous study, Yu et al. [47] examined the subcellular localization of hUTP4/Cirhin by making an extensive panel of hUTP4/Cirhin protein fragments and concluded that hUTP4/Cirhin contains a nucleolar localization signal between amino acids 315–432. However, the K514X and D557X mutants presented in this study both contain this putative nucleolar localization signal but do not localize to the nucleolus. The presence of this sequence may therefore not be sufficient for nucleolar localization. Rather, we suggest that the interaction between hUTP4/Cirhin and NOL11 may be required for proper subcellular localization of hUTP4/Cirhin, a hypothesis that is supported by our observation that hUTP4/Cirhin mutants that fail to interact with NOL11 also fail to localize to the nucleolus.

To analyze the interaction between hUTP4/Cirhin and NOL11, we made a panel of hUTP4/Cirhin mutants. C-terminal truncations of hUTP4/Cirhin demonstrated reduced interaction with NOL11, but an N-terminal deletion persisted in interaction, confirming that NOL11 does indeed interact with the region of hUTP4/Cirhin containing the NAIC mutation. Interestingly, Y2H analysis also revealed that NOL11 interacts with full-length hUTP4/Cirhin containing the R565W mutation, but has greatly reduced interaction with a C-terminal fragment of hUTP4/Cirhin containing the R565W mutation. Therefore, our studies provide evidence that the pathogenesis of NAIC lies in a defective protein-protein interaction between two proteins required for ribosome biogenesis. Given that full-length hUTP4/Cirhin containing the NAIC mutation still interacts with NOL11, it is likely that the NAIC mutation causes only a partial loss of interaction between hUTP4/Cirhin and NOL11 in patients. This result is expected since hUTP4/Cirhin is essential in mammals [48] and complete loss of interaction between hUTP4/Cirhin and NOL11 would likely completely disrupt ribosome biogenesis leading to embryonic lethality.

Within the past decade, multiple diseases of ribosome biogenesis, the so-called “ribosomopathies”, have been described and all of them appear to be the result of haploinsufficiency or other partial loss of ribosome biogenesis or ribosome function [reviewed in 49]. Two of these diseases, Treacher Collins syndrome [50] and CHARGE syndrome [51] are caused by mutations in proteins that are required for optimal rDNA transcription. Others, including Bowen-Conradi syndrome [52] and ANE syndrome [53], are caused by missense mutations in ribosome biogenesis proteins. Similarly, recent studies on proteins that share structural similarities to hUTP4/Cirhin (all contain WD40 repeats) have shown that missense mutations in WDR11 or WDR62 can cause disease either by reduced interaction with a binding partner [54] or by improper subcellular localization [55], respectively. Similarly, here we have shown that the NAIC mutation does indeed cause a partial loss of hUTP4/Cirhin interaction with NOL11 in the context of a hUTP4/Cirhin fragment. Our results provide evidence that the pathophysiology of NAIC is, at least in part, caused by a defective hUTP4/Cirhin – NOL11 protein-protein interaction.

## Materials and Methods

### Plasmids

A full-length hUTP4/Cirhin cDNA clone was purchased from GeneCopoeia. Full-length hUTP4/Cirhin or a C-terminal fragment of hUTP4/Cirhin was amplified by PCR and then subcloned into the pGBK vector (Clontech) between the EcoRI and SalI restriction sites. Mutations in pGBK-hUTP4/Cirhin were introduced using a Change-IT kit (USB Corporation) and oligonucleotides containing the desired mutations per the manufacturer's instructions. Mutations in hUTP4/Cirhin were also introduced into the Gateway Destination vector pcDNA6.2/nLumio-DEST (Invitrogen) construct using a QuikChange II XL Site-Directed Mutagenesis Kit (Stratagene). All mutations were confirmed by sequencing. A full-length cDNA clone of NOL11 was purchased from Open Biosystems and subcloned into the Gateway Entry vector pDONR221 (Invitrogen). It was then transferred by LR reaction into pASGW [56]. The NOL11 ORF was also LR transferred into pcDNA6.2/nLumio-DEST (Invitrogen). The pHrD-IRES-Luc plasmid was a gift from Dr. Yang Ke (Peking University, Beijing, China) and the *Renilla* plasmid was a gift from Dr. Joan Steitz (Yale University, New Haven, CT).

### Yeast two-hybrid analysis

hUTP4/Cirhin was cloned into the pGBK bait vector (*TRP1* marker, kan^R^; Clontech) and transformed into the Y2HGold yeast strain (*MATa, trp1-901, leu2-3, 112, ura3-52, his3-200, gal4Δ, gal80Δ, LYS2::GAL1_UAS_–Gal1_TATA_–His3, GAL2_UAS_–Gal2_TATA_–Ade2, URA3::MEL1_UAS_–Mel1_TATA_ AUR1-C MEL1*; Clontech). Yeast containing pGBK-hUTP4/Cirhin were mated to yeast containing a cDNA prey library derived from human liver (Mate & Plate Library, pGAD vector, *LEU2* marker, Amp^R^; Clontech) per the manufacturer's instructions. Yeast were then plated on medium lacking tryptophan, leucine, and histidine, and containing 40 µg/ml 5-Bromo-4-Chloro-3-indolyl-α-D-galactopyranoside (X-α-gal) and 3 mM 3-Amino-1,2,4-triazole (3-AT) (D-W-L-H+X-α-gal+3-AT). Plates were incubated at 30°C for 2 weeks. Growth of a blue yeast colony on this medium indicates a positive protein-protein interaction. Prey plasmids from positive colonies were recovered as follows: total DNA was extracted from yeast by the phenol/chloroform/isoamyl alcohol method and then transformed into *E. coli*. These cells were plated on medium containing carbenicillin to select specifically for the prey vector. All prey clones were sequenced by the DNA Analysis Facility on Science Hill at Yale University.

For directed yeast two-hybrid analysis, all proteins were cloned into both bait (pGBK, *TRP1* marker; Clontech) and prey (pACT2, *LEU2* marker; Clontech) vectors. Both bait and prey vectors were transformed sequentially into the Y2HGold yeast strain and plated on medium lacking tryptophan and leucine. Yeast that grew on medium lacking tryptophan and leucine were then transferred to D-W-L-H+X-α-gal plates and incubated at 30°C for 1 week to assay for protein-protein interactions.

### Protein purification and mass spectrometry

hUTP4/Cirhin was cloned into the pN-TGSH plasmid (Dualsystems), to create an N-terminal Strep-HA fusion. This plasmid was then introduced into the Flp-In 293 T-REx cell line (Invitrogen) to generate cells that stably express Strep- and HA-tagged hUTP4/Cirhin. Expression of hUTP4/Cirhin was confirmed by Western blot with an anti-HA antibody. hUTP4/Cirhin and associated proteins were isolated by double affinity purification and then subjected to trypsin digestion. The peptide mixture was separated on a C18 HPLC column and analyzed by direct LC/MS-MS using an LTQ Orbitrap XL mass spectrometer (Thermo Fisher Scientific). Peptides were identified using the Human International Protein Index (IPI) protein database. Creation of the cell line, protein purification and mass spectrometry were performed by Dualsystems Biotech (Schlieren, Switzerland).

### Protein alignments

Orthologs of NOL11 were identified by a BLASTp search of the GenBank non-redundant protein database (http://blast.ncbi.nlm.nih.gov/Blast.cgi) using human NOL11 (NP_056277.2) as a query. Orthologs were identified in the following species: *Pan troglodytes* (XP_001164856.1), *Mus musculus* (NP_598463.2), *Monodelphis domestica* (XP_001370403.1), *Xenopus laevis* (NP_001085113), *Danio rerio* (NP_001025234.1), *Aedes aegypti* (XP_001661381.1), *Drosophila melanogaster* (NP_649127.1), *Trichoplax adhaerens* (XP_002109520.1), and *Capsaspora owczarzaki* (EFW43073.1). Sequence alignments and percent identity/similarity were determined with BioEdit [57].

### Cell culture and RNAi

HeLa cells and HT1080-3D1 cells were grown in Dulbecco's Modified Eagle Medium (DMEM; GIBCO) supplemented with 10% fetal bovine serum. HT1080-3D1 cells were additionally supplemented with 50 U/mL penicillin/streptomycin and 5 µg/mL Blasticidin. MCF-10A cells were grown in DMEM supplemented with 5% horse serum, 20 ng/mL EGF, 0.5 µg/mL hydrocortisone, 100 ng/mL Cholera toxin, 10 µg/mL insulin and 5 U/mL penicillin/streptomycin. All cells were incubated at 37°C in a humidified atmosphere with 5% CO_2_. To knock down hUTP4/Cirhin or NOL11, siRNA SMARTpools (Dharmacon) were transfected into cells at a final concentration of 20 nM. A non-targeting siRNA (Dharmacon, #1) and mock transfection were also included. HeLa cells were seeded in either 24-well, 12-well, or 6-well plates. Cells were transfected the following day using 0.25 µl, 0.5 µl, or 1 µl of DharmaFECT1 transfection reagent per well of a 24-well, 12-well, or 6-well plate, respectively. For complete knockdown of NOL11, cells were transfected a second time after a further 48 h. Knockdown of hUTP4/Cirhin and NOL11 was assessed by Western blot 48 h after the first or second round of transfection, respectively.

### Immuno-fluorescent cell staining and imaging

HT1080-3D1 cells were fixed with ice cold methanol for 10 min at −20°C and then washed 3 times with PBS. ActD treated cells were treated with 0.1 µg/ml ActD for 1 hr prior to fixation. Immunofluorescence staining for NOL11 was carried out using an anti-NOL11 antibody (Novus Biologicals, NBP1-90522) at a 1∶200 dilution. Immunofluorescence staining for UBF was performed as previously described [27].

Z-stacks of fluorescent images were captured using a Photometric Coolsnap HQ camera and Volocity 5 imaging software (Improvision, PerkinElmer) with a 63× Plan Apochromat Zeiss objective mounted on a Zeiss Axioplan2 imaging microscope. Extended focus images of deconvolved Z-stacks (iterative restoration) are illustrated.

To image nucleoli after siRNA treatment, an anti-fibrillarin antibody (72B9) [30] was used at a 1∶2500 dilution. Cells were imaged at 60× magnification using the Opera High Content Screening System and Acapella High Content Imaging and Analysis Software (PerkinElmer). siRNA treatment and imaging for nucleolar morphology assays were carried out at the Yale Center for High Throughput Cell Biology (Connecticut, USA).

### Western blotting and co-immunoprecipitations

For Western blotting from human cell lines, HeLa cells were harvested by adding 300 µl 1× lysis buffer (50 mM Tris-HCl pH 6.8, 100 mM DTT, 2% SDS, 0.1% Bromophenol blue, 10% glycerol) directly to each well of a 6-well dish after cells were washed once with 1× PBS. Cells were then lysed by passage seven times through a 27 1/2 G needle. For Western blotting from yeast cells, 5 ml of cells at an OD_600_ of ∼1 were collected from yeast cultures expressing a bait protein (pGBK vector) and cells were lysed by the post-alkaline extraction method [58]. For both human and yeast cells, whole cell extract was loaded onto 6% or 10% polyacrylamide gels and proteins were separated by SDS-PAGE. Proteins were transferred to a PVDF membrane (Millipore) and were detected using one of the following antibodies: anti-hUTP4/Cirhin (Abnova Corporation, H00084916-A01) at a 1∶2000 dilution, anti-NOL11 (Sigma, HPA022010) at a 1∶2000 dilution, anti-β-actin (Sigma, A1978) at a 1∶40000 dilution, anti-MYC (9E10 ascites) [39] at a 1∶1000 dilution, or anti-Mpp10 [59] at a 1∶10000 dilution.

For immunoprecipitations, HeLa cells were harvested by adding 650 µl NET2 (20 mM Tris-HCl pH 7.5, 150 mM NaCl, 0.05% Nonidet P-40) to a 10 cm dish after cells were washed once with 1× PBS. One 10 cm dish of cells was harvested per sample. ActD treated cells were treated with 0.1 µg/ml ActD for 1 hr before cells were harvested. Protease inhibitors (Roche) were added to the harvested cells and then cells were lysed by sonication for three 30 s periods with 1 min on ice in between. Total lysates were cleared by centrifugation at 20,000 *g* for 10 min at 4°C. Anti-fibrillarin antibody (72B9, 100 µl per sample) was conjugated to protein A-Sepharose CL-4B beads (3 mg per sample) by incubation on a nutator overnight at 4°C. Antibody-bound beads were washed three times in NET2 and then incubated for 1 h at 4°C with 650 µl cleared cell lysate per sample. Beads were then washed 5 times with NET2 to remove unbound protein. Total protein (5%) and immunoprecipitations were separated by 10% SDS-PAGE and then transferred to a PVDF membrane. Western blotting was then performed as described above.

### Northern blotting

HeLa cells were siRNA treated or mock treated as described above. Cells were then harvested in TRIzol (Invitrogen) and total RNA was extracted per the manufacturer's instructions. Northern blot analysis was performed similarly to previously described [60]. Briefly, 3 µg of RNA per sample was separated by electrophoresis on a 1% agarose/1.25% formaldehyde gel and then transferred to a nylon membrane (Hybond-XL, GE Healthcare). RNA species were detected by hybridization with radiolabelled oligonucleotide probes or by methylene blue staining. Oligonucleotide probes are as follows: a, 5′-CCT CTC CAG CGA CAG GTC GCC AGA GGA CAG CGT GTC AGC-3′ and c, 5′-AAG GGG TCT TTA AAC CTC CGC GCC GGA ACG CGC TAG GTA C-3′.

### Luciferase assay

HeLa cells were siRNA treated or mock treated as described above. Twenty-four hours before harvesting, the cells were transfected with 400 ng pHrD-IRES-Luc [31] and 1 ng of a *Renilla* containing plasmid [34] using X-tremeGENE 9 (Roche). Luciferase activities were measured using a 20/20n luminometer (Turner BioSystems) and the Dual Luciferase Assay System (Promega) per the manufacturer's instructions. To control for transfection efficiency, the ratio of pHrD-IRES-Luc activity:*Renilla* activity was calculated. The ratios for each siRNA were then normalized to their respective mock transfections and these normalized values were plotted.

## Supporting Information

Table S1 Full list of proteins that co-purify with hUTP4/Cirhin as identified by mass spectrometry.
(DOC)
