# Supplementary material for: NOL11, Implicated in the Pathogenesis of North American Indian Childhood Cirrhosis, Is Required for Pre-rRNA Transcription and Processing
Source: PLoS Genet. 2012 Aug 16;8(8):e1002892. doi: 10.1371/journal.pgen.1002892 (PMC3420923; doi:10.1371/journal.pgen.1002892)
Supplement: Table S1 — Full list of proteins that co-purify with hUTP4/Cirhin as identified by mass spectrometry. (DOC) [file pgen.1002892.s001.doc]

Table S1. Full list of proteins that co-purify with hUTP4/Cirhin as identified by mass spectrometry.

| Class^+^ | Gene name | Alias | Swissprot Accession | Unique peptides  in experiment 1 | Unique peptides  in experiment 2 |
| --- | --- | --- | --- | --- | --- |
| Bait | hUTP4 | CIRH1A | Q969X6 | 44 | 40 |
| A | hUTP5 | WDR43 | Q15061 | 13 | 11 |
| A | NOL11 |  | Q9H8H0 | 8 | 4 |
| A | UTP15 |  | Q8TED0 | 7 | 3 |
| A | ACACA* |  | Q13085 | 6 | 8 |
| A | hUTP17 | WDR75 | Q8IWA0 | 3 | 4 |
| B | MASP1 |  | P48740 | 2 | 1 |
| B | WDR81 |  | Q562E7 | 1 | 1 |
| B | H1F0* |  | P07305 | 1 | 1 |
| C | HIST1H1C* |  | P16403 | 9 | 0 |
| C | NONO* |  | Q15233 | 2 | 0 |
| C | EWSR1* |  | Q01844 | 2 | 0 |
| C | HBA1* |  | P69905 | 2 | 0 |
| C | PLIN4 |  | Q96Q06 | 1 | 0 |
| C | RNF20 |  | Q5VTR2 | 1 | 0 |
| C | PAFAH1B2 |  | P68402 | 1 | 0 |
| C | GLUD2 |  | P49448 | 1 | 0 |
| C | AMBRA1 |  | Q9C0C7 | 1 | 0 |
| C | INPP5D |  | Q92835 | 1 | 0 |
| C | S100A7 |  | P31151 | 1 | 0 |
| C | H1FX* |  | Q92522 | 1 | 0 |
| C | SYNE1 |  | Q8NF91 | 0 | 1 |
| C | FLJ46204 |  | Q6ZRP5 | 0 | 1 |
| C | SMOX |  | Q9NWM0 | 0 | 1 |
| C | HNRNPR* |  | O43390 | 0 | 1 |
| C | DAAM2 |  | Q86T65 | 0 | 2 |

+ Bait: The protein that was tagged for affinity purification

A: Proteins that were identified in both biological duplicates and had at least 5 unique peptides identified between duplicates. These proteins are highly likely interactors.

B: Proteins that were identified in both biological duplicates. These proteins are likely interactors.

C: Proteins that were identified in only one biological duplicate. These proteins may represent transient and/or substoichiometric interactors.

* Proteins that are repeatedly isolated with other bait proteins
